# DyHAP: Dynamic Hybrid ANFIS-PSO Approach for Predicting Mobile Malware

**DOI:** 10.1371/journal.pone.0162627

**Published:** 2016-09-09

**Authors:** Firdaus Afifi, Nor Badrul Anuar, Shahaboddin Shamshirband, Kim-Kwang Raymond Choo

**Affiliations:** 1 Faculty of Computer Science and Information Technology, University of Malaya, Kuala Lumpur, Malaysia; 2 Department of Information Systems and Cyber Security, University of Texas at San Antonio, San Antonio, Texas. United States of America; 3 School of Information Technology & Mathematical Sciences, University of South Australia, Adelaide, South Australia, Australia; 4 School of Computer Science, China University of Geosciences, Wuhan, China; Beihang University, CHINA

## Abstract

To deal with the large number of malicious mobile applications (e.g. mobile malware), a number of malware detection systems have been proposed in the literature. In this paper, we propose a hybrid method to find the optimum parameters that can be used to facilitate mobile malware identification. We also present a multi agent system architecture comprising three system agents (i.e. sniffer, extraction and selection agent) to capture and manage the pcap file for data preparation phase. In our hybrid approach, we combine an adaptive neuro fuzzy inference system (ANFIS) and particle swarm optimization (PSO). Evaluations using data captured on a real-world Android device and the MalGenome dataset demonstrate the effectiveness of our approach, in comparison to two hybrid optimization methods which are differential evolution (ANFIS-DE) and ant colony optimization (ANFIS-ACO).

## Introduction

The ubiquity and popularity of mobile devices is likely to increase in the foreseeable future. For example, according to the Global Web Index, 80% of Internet users own at least a smartphone and the online mobile shopping showed 150% increase in 2015 compared to 2014 [1]. Due to the widespread use of mobile devices and the amount of personal information stored on these devices, they have become the targets of cybercriminals such as malware authors and hackers [2–5]. Android devices are one of the most targeted platforms due to its market share, and open nature of the operating system [6–9]. One popular mitigation strategy used by mobile device users is anti-malware app. However, a recent systematic evaluation of popular free Android cloud-based anti-malware apps concluded:

*that no single cloud anti-malware app can be solely relied upon to mitigate known malware*. *The findings were also concerning*, *particularly that malware threats are becoming more sophisticated and targeted*, *using various attack vectors to escalate permissions and exfiltrate data* [10]

Not surprisingly, mobile security and malware detection has been the subject of recent research. In order to detect malware, one could deploy an intrusion detection system (IDS) which can be either anomaly-based or signature-based (also called behavioral-based). The signature-based approach relies on a predefined pattern or malware signature. While such approach is popular, they are ineffective in detecting unknown malware [11,12]. Unlike signature-based detection, the anomaly-based approach seeks to differentiate between normal and abnormal conditions. For example, abnormal conditions include specific malware characteristics (e.g. malware code and its logical structure) or behaviors, identified during the analysis of such applications (i.e. malware analysis).

Malware analysis, a process of understanding how a particular piece of malware functions by dissecting and studying the code and its behavior with the aims of mitigating the threat [13], can be broadly categorized into static or dynamic analysis. Techniques such as machine learning have been utilized to differentiate normal and abnormal patterns in suspicious applications. For example, Dimitrios *et al*. [14] evaluated the suitability of five machine learning classifiers, namely: Radial Basis Function (RBF), Bayesian Networks, K-Nearest Neighbors (KNN) and Random Forest in detecting anomalies on mobile devices. Similarly, Feizollah *et al*. [15] analyzed the performance of machine learning classifiers in detecting Android malware and findings as high as 99.94% detection rate for KNN. Despite the amount of efforts on the topic, mobile malware detection remains a topic of active research (and the focus of this paper).

In recent times, a number of studies have seek to evaluate the effectiveness of computational intelligence-based solution for improved performance [16]. For example, FUGE [17] uses fuzzy theory and genetic method in cloud job scheduling algorithm. Findings from the authors’ evaluations suggested the approach is efficient in terms of execution time, execution cost, and average degree of imbalance. Similarly, FR-TRUST [18] uses fuzzy theory to compute a peer trust level, and has been demonstrated to provide a high—ranking accuracy. In the study of malware, however, there are relatively few research works that use the fuzzy inference system because this is a complex NP problem. It is unlikely that efficient algorithms for solving this problem are deterministic; hence, the interest in heuristic algorithms.

We introduce an integrated method to detect mobile malware in this paper. Specifically, we use neural network function and regression to generalize the relationships between inputs based on the Adaptive Neuro-Fuzzy Inference System (ANFIS). The particle swarm optimization was also combined in our approach in order to optimize the malware prediction model. We then seek to evaluate the effectiveness of our approach and compare its performance with other hi-tech soft computing hybrid approaches.

The rest of this paper is structured in sections as follows: review of the related work (see Section 2), research methodology (see Section 3), proposed ANFIS framework (see Section 4), and evaluation of findings (see Section 5). Finally, the last section concludes this study.

## Related Work

Malware detection approaches are categorized into anomaly-based and signature-based detection [19]. The signature-based method finds malware by comparing collected information from monitored users and system activities to an existing list of known malicious files database (i.e. malware signatures) [20]. While this approach has worked in the past, it is largely ineffective against new malware whose signature does not yet exist, or malware that uses “oligomorphic”, “polymorphic” and “metamorphic” to avoid detection by encrypting or modifying parts of the code [21]. Such an approach also requires user to constantly update the signature database. Anomaly-based approach monitors and analyzes network traffic, system, user activity levels, etc. for a particular pattern of behavior. An intrusion is flagged when there is a deviation from the normal behavior patterns [22]. Machine learning classifiers, such as support vector machine, neural network, genetic algorithm, fuzzy logic, and decision tree, have been used in malware detection models [23]. To optimize malware detection model, selection of particular features is important in the machine learning classification process.

Malware analysis can be static or dynamic [24]. In static analysis, a program is examined by inspection without execution the actual application. Such process is normally performed manually by malware analysts to understand the logical structure, flow and data content stored within the binary itself, behavior of the suspicious application, etc. [25,26], [27]. For instance using Android Application Package (APK) file, [28] and [29] used file permissions as key reference points to detect malware on Android devices. However, Android malware such as DroidKungFuUpdate can avoid from being detected by not requesting access to suspicious permissions [30]. Do, Martini and Choo [31] in a recent work demonstrated how data can be exfiltrated from an Android device using inaudible sound waves via the device’s speaker, which requires no permission. With the constant evolution of (mobile) malware and significant increase in the number of applications, it would be impossible to manually analyze all suspicious applications. In dynamic analysis, application activities such as network traffic and system calls are analyzed while the application is running. For example, Crowdroid [32] collected the device’s kernel system calls to determine the application patterns. However, collecting system calls is a complicated task which requires device to be rooted. This can result in devices being more vulnerable to malicious exploitation. Yerima *et*. *al* [33] analyzed requested permissions of 2000 applications and determined that more than 93% of malware applications request for network connectivity (e.g. to communicate with the command and control server and to exfiltrate data). This indicated that, malicious applications tend to use network more than normal applications. Hence, we focus on analyzing mobile network traffic.

A variable selection process through the ANFIS was used to find the most significant parameters in malware detection. The aim was to find a subset of the logged variables that shows good prognostic abilities [34–36], and one can filter irrelevant variables by use of former knowledge. Donald A. [37] proposed genetic algorithm (GA) based variable selection for optimization, which aim to decrease the error between true values and prediction model by choosing the suitable explanatory variables (input). the ANFIS [38,39] was employed as a powerful tool for the variable selection in this paper. ANFIS has also been used in several engineering fields for modelling [40–43], predictions [44–46] and control [47–50]. The main idea of neuro-adaptive learning methods is to perform the fuzzy modelling procedure for data learning [51,52]. The ANFIS forms the fuzzy inference system with pairs (input/output) of data [53]. This approach enables fuzzy logic to adapt the membership function parameters to best track the given input/output data by the fuzzy inference system.

Metaheuristic optimization algorithms have become popular choice for solving complex problem [54]. As pointed out by one of the reviewers that combining ANFIS and Particle Swarm Optimization (PSO) for prediction problems has been widely studied and understood [55–57]. Pooranian and Shojafar [58], for example, proposed combining PSO with the gravitational emulation local search (GELS) to solve the independent task scheduling problem in grid computing. Jiang also proposed a PSO based ANFIS approach to improve accuracy in modelling customer satisfaction, and demonstrated that such an approach achieves better performance than fuzzy regression (FR), ANFIS and Genetic Algorithm (GA) based ANFIS approaches [59]. Other combinations of PSO and ANFIS have been proposed in forecasting such as in short-term wind power [60], and spur dike’s parameters [61]. In order to increase its accuracy and performance, this paper applied three optimization techniques to ANFIS which are ANFIS-PSO (ANFIS-particle swarm optimization), ANFIS-DE (ANFIS-differential evolutionary) and ANFIS-ACO (ANFIS-ant colony optimization). These hybrid algorithms help improve the ANFIS performance by tuning the membership function towards zero error analysis.

## Research Methodology

This section describes our experiment setup, which consists of two phases, namely: data collection, and feature selection, extraction and labelling phase.

### Data Collection

**Fig 1:**
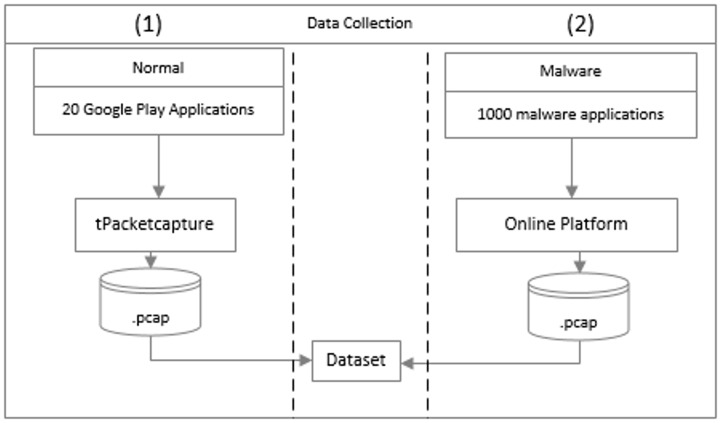
Data collection phase.

We gathered and analyzed network traffic developed by Android apps. In this phase, different approaches were used to capture malware and normal network traffic (Fig 1).

Twenty popular (and reputable) apps from four different app categories were downloaded from Google Play and installed on a mobile device running Android operating system Jelly Bean version 4.1.2 (see Table 1). Prior to installation, we checked the authenticity of the apps. The network traffic from running these apps was captured in a real-time network environment, where each app was run for 30 minutes.

**Table 1:** Normal application categorization.

App	Total	Description
Social	3	Enables user to interact with other users or find people who share common interest such as hobbies, religion, politics, and alternative lifestyles
Communication	6	Enables user to make (free) phone call, video call, send multimedia message, attach file using network connection
Game	10	Enables user to play for enjoyment with certain situation either for educational or amusement purpose. It can be grouped with network connection or connected with social website.
Tool	1	Enables user to customize phone feature

Of the 1260 malware data samples from 49 families in the Malgenome [30] dataset, we captured the network patterns of 1,000 samples. The samples were analyzed in real-time with public malware-detection sandbox, namely: Anubis Iseclab [62] and automatic Android program analysis, SanDroid [63], since the malware data samples in the dataset were generated by these platforms.

### Feature Selection, Extraction and Labelling

**Fig 2:**
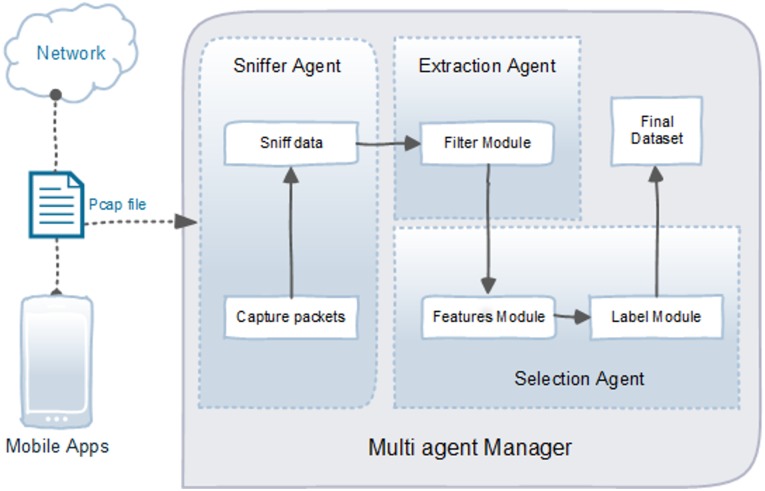
Multi agent.

In this multi agent system architecture (Fig 2), three system agent is proposed to capture and manage the pcap file for data preparation phase. The details of each agent and its role are given in the next section.

#### Sniffer Agent

**Fig 3:**
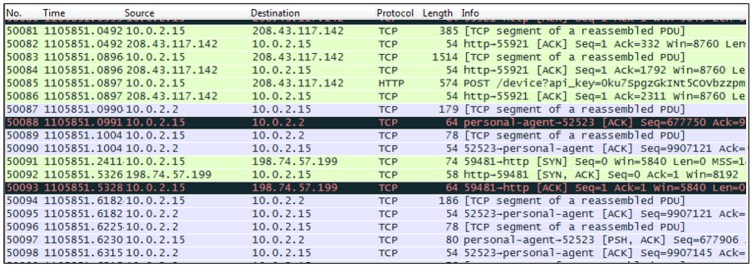
Sample of captured packets.

In the sniffer agent, the pcap file is capture from the network connection between mobile apps and internet. Sniffer module using tShark (a network protocol analyzer) [64], to retrieved all related information (see Fig 3). Later the sniffed data is fed to extraction agent.

#### Extraction Agent

**Fig 4:**
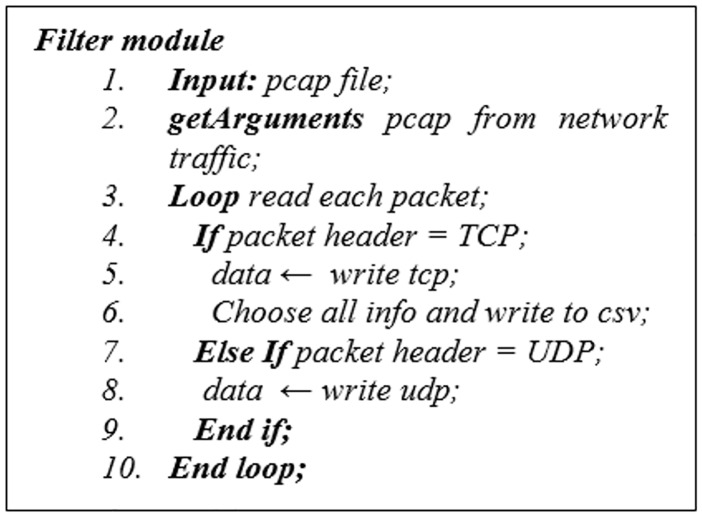
Pseudocode of filter module.

Extraction agent consist of filter module which filter the collected network traffic using Java and Wireshark routine to clear the captured packets from unwanted data. For example, we only use TCP packets in the network traffic data and remove UDP and *Domain name system* (DNS) packets from it. The pseudocode of this module is shown in Fig 4.

#### Selection Agent

This agent is one of the most important agents in this model. This agent has two modules which is Feature Module and Label Module. Feature Module choose a number of features to be used as main attributes to classify mobile malware. The features were chosen from a wide range of features in unbiased packet level features of the TUIDS intrusion dataset. The main challenge in this phase was to identify the best applicable features in particular, which result in higher detection accuracy and avoiding an overfitting model. The dataset needs to be filtered and refined from numerous excessive features. Some of the features are linked, which can complicate the process of malware detection. Furthermore, features with redundant information from other features may reduce the detection model accuracy and increase computational time and complexity of the model. In this study, a specific method to select the best attributes from machine learning tool Weka [65] called ClassifierSubsetEval was applied.

**Fig 5:**
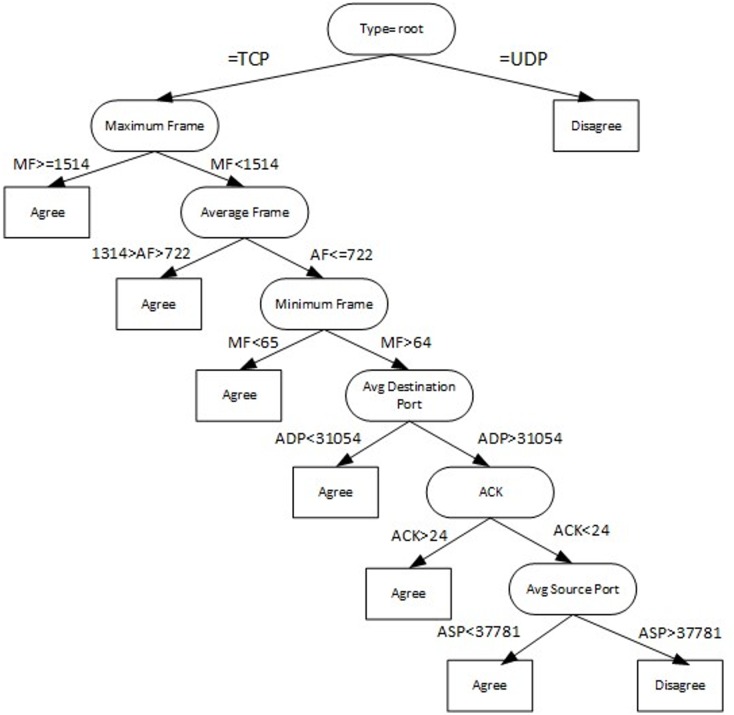
Decision tree for data labeling.

We choose seven connection-based features to analyze, as shown in Table 2. The extracted features were stored as a sequence of comma separated values (CSV) file. Next, after dataset was extracted with selected features, it was passed to Label module which labeled the dataset according to Fig 5. This phase remove noise in dataset and to ensure experiment validity. The final dataset from a combination of normal and infected data consists of three hundred thousand rows of data with seven features, prior to splitting into 70% training and 30% testing dataset. In order to avoid overfitting issue, we train our model with a wide range of examples and split datasets.

**Table 2:** Input and output parameters.

Inputs/Output	Parameters	Description
**input 1**	Maximum_Frame	The maximum number of frame in last P packets.
**input 2**	Frame_STD	Standard Deviation for frame in P packets
**input 3**	Count_ACK	The number of Acknowledge packet in the last P packets.
**input 4**	Minimum_Frame	The minimum number of frame in last P packets.
**input 5**	Average_Dest_Port	Average number of unique destination port in the last P packets.
**input 6**	Average_Frame	The average frame flowing in the last P packets.
**input 7**	Average_Source_Port	Average number of unique source port in the last P packets.
**output 1**	0,1	Uninfected = 0, Infected = 1

## Proposed Approach

We introduce an approach in this paper that combines adaptive neuro fuzzy inference system (ANFIS) and particle swarm optimization (PSO). We used PSO to improve performance of ANFIS by adjusting the membership functions and minimizing the error. Forecasts from ANFIS can be used to reconstruct future behavior of the malware.

### Particle swarm optimization (PSO)

PSO is an approach for optimizing “continue” and “discontinue” decision making functions, which develop by Dr. Kennedy and Dr. Eberhart in 1995 [55]. PSO has been used to model animals’ sociological and biological behavior (like groups of birds searching for food) [66]. The PSO has also been employed in population-based search approach, in which a particle of a population is present for each individual potential solution or swarm. In this method, the position of particle is changed constantly in a search space until reaching to the optimum solutions and computational restrictions are reached.

Former experiential research shows the efficiency and advantages of the mentioned method for optimization [67], [68].

For example, in an optimization issue with D variables, a swarm of N particles is established in a way that every particle will be allotted to an arbitrary position in the hyperspace with D measurements. Position of each particle for this situation is associated with a possible answer for the optimization matter. Both *v* and *x* are flight speed of a particle over a solution space and its position (direction). A scoring capacity is allocated to every individual *x* in the swarm, which gains a wellness value. The latter is an indication of its competence to address the issues.

A particle’s best prior position is represented by Pbest, and Gbest signifies the best swarm particle. Each particle can log its own Pbest and find its Gbest. Subsequently, all particles that move over the D-dimensional solution space should follow the rules updated for new positions until they achieve optimum position. The subsequent deterministic and stochastic update rules show how a particle’s position and velocity are updated (Eq 1):

$$v_{i}\left(t\right)=\omega v_{i}\left(t-1\right)+\rho_{1}\left(x_{Pbest_{i}}-x_{i}\left(t\right)\right)+\rho_{2}\left(x_{Gbest}-x_{i}\left(t\right)\right)$$ (1)

$$x_{i}\left(t\right)=x_{i}\left(t-1\right)+v_{i}\left(t\right)$$ (2)

In the above equation, random variables are shown by *q*_*1*_ and *q*_*2*_ and *x* represents an inertia weight.

Positive acceleration constants are represented by C_1_ and C_2_ and the random variables are outlined as *q*_1_ = *r*_1_*c*_1_ and *q*_2_ = *r*_2_c_2_, with (*r*_1_, *r*_2_, *U*(0,1)). The stochastic and weights of growing speed terms that lead to a particle reaching to the Gbest and Pbest have speeding up constants of C_1_ and C_2_. A particle can move a long distance from the target locales when the qualities are few, while huge qualities cause the abrupt particles development to target locales. In line with the average practice in [69], both C_1_ and C_2_ constants are equal to 2.0 in this study. In Eq 2, the best likely amendment of dormancy x provides a harmony between the nearby and worldwide examinations, which reduces the amount of emphases on finding an ideal arrangement. A latency rectification capacity called the IWA or “idleness weight approach” was used in this exploration work [69,70]. The x (latency weight) is changed amid the IWA according to the associated relationship:

$$\omega =\omega_{\text{max}}-\frac{\omega_{\text{max}}-\omega_{\text{min}}}{{\text{Itr}}_{\text{max}}}Itr$$ (3)

In Eq 3, x_max_ and x_min_ represent the primary and ultimate inertia weights, the current number of iteration is represented by Itr and the maximum number of iteration is represented by Itr_max_.

### ANFIS

**Fig 6:**
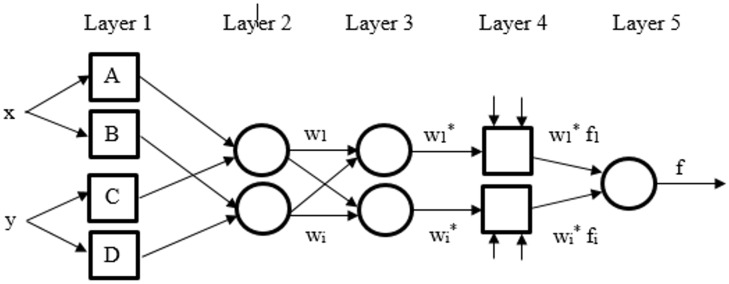
ANFIS structure.

The term adaptive neuro-fuzzy inference system was introduced by Jang, 1993 refer to combination of Fuzzy Logic and Artificial Neural Network to produce a powerful processing tool [71]. For every input, two fuzzy if-then rule were generate in this study with maximum equal to 1 and minimum equal to 0. Fig 6 shows the ANFIS arrangement and inputs.

Assume two inputs fuzzy if-then rules of Takagi and Sugeno’s type [72] were adopted:

$$if\;i\;is\;A\;and\;j\;is\;C\;and\;k\;is\;E\;and\;l\;is\;G\;then\;f_{1}=p_{1}i+q_{1}j+r_{1}k+s_{1}l+t$$ (4)

Layer 1 contains membership functions (MFs) of input variables and feed input values for the next layer. Each node in 1^st^ layer is adaptive as: *o* = *μ*(*i*), where *μ*(*i*)_*i*_ are membership functions.

**Fig 7:**
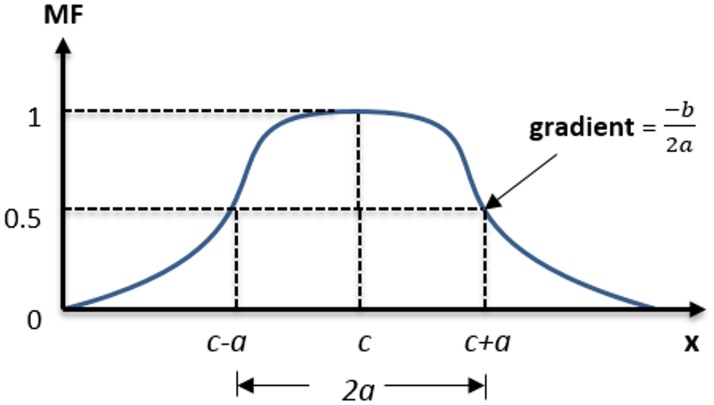
Three parameters in bell membership function; (a, b and c).

The bell-shaped membership functions (Fig 7) is presented in Eq 5 for which the lowest and highest amounts are 0 and 1, respectively.

$$f\left(x;a,b,c\right)=\frac{1}{1+{\left(\frac{x-c}{a}\right)}^{2b}}$$ (5)

The function is subject to the following parameters, namely *a*, *b* and *c*. Each of these parameters define as follows: *a* is half width of the curve; *b* defines the gradient together with *a*; and *c* is the midpoint of the membership function as shown in Fig 7.

In the 2nd layer (the membership layer), the weight of MFs is considered. The first layer provides the input values for layer 2. The nodes in the second layer are fixed node. The output is the product from all incoming signals and be described as,

$$w_{i}=\mu {\left(i\right)}_{i}\cdot \mu {\left(i\right)}_{i+1}$$ (6)

Output of every node indicates the weight strength of a rule.

In layer 3 which is the rule layer, every node does the pre-condition matching of the fuzzy rules, that calculate each rule’s activation level as well as the normalized firing strength. This is a fixed layer as well, and each node computes the proportion of *i*th rule of the firing strength to the sum of *i*th firing strengths of all rules as:

$$w_{i}^{*}=\frac{w_{i}}{w_{1}+w_{2}},\;\;for\;\;i=1,2$$ (7)

The outputs of this layer are named as normalized weights or firing strengths.

In layer 4 or defuzzification layer, all the adaptive nodes provide the resulting output values from the inference of rules.

$$O_{i}^{4}=w_{i}^{*}\cdot f=w_{i}^{*}p_{1}i+q_{1}j+r_{1}k+s_{1}l+t$$ (8)

Here, the parameters set is shown as {*p*_i_, *q*_i_, *r*_*i*_, *s*_*i*_, *t*}.

Layer 5 or the output layer summarizes the inputs output from layer 4. This layer also transforms the results of fuzzy classification into a crisp. Here, the single node is fixed node and the whole incoming signals is sum up to produce overall output as below,

$$O_{i}^{5}=\underset{i}{\sum }w_{i}^{*}\cdot f=\frac{\sum_{i}w_{i}\cdot f}{\sum_{i}w_{i}}$$ (9)

The PSO method was used in this paper to help ANFIS adjust the membership function parameters [70]. The main advantage of PSO technique is its friendly way of calculation in a network topology of given size. The membership functions were triangular in this study.

### ANFIS-PSO algorithm

**Fig 8:**
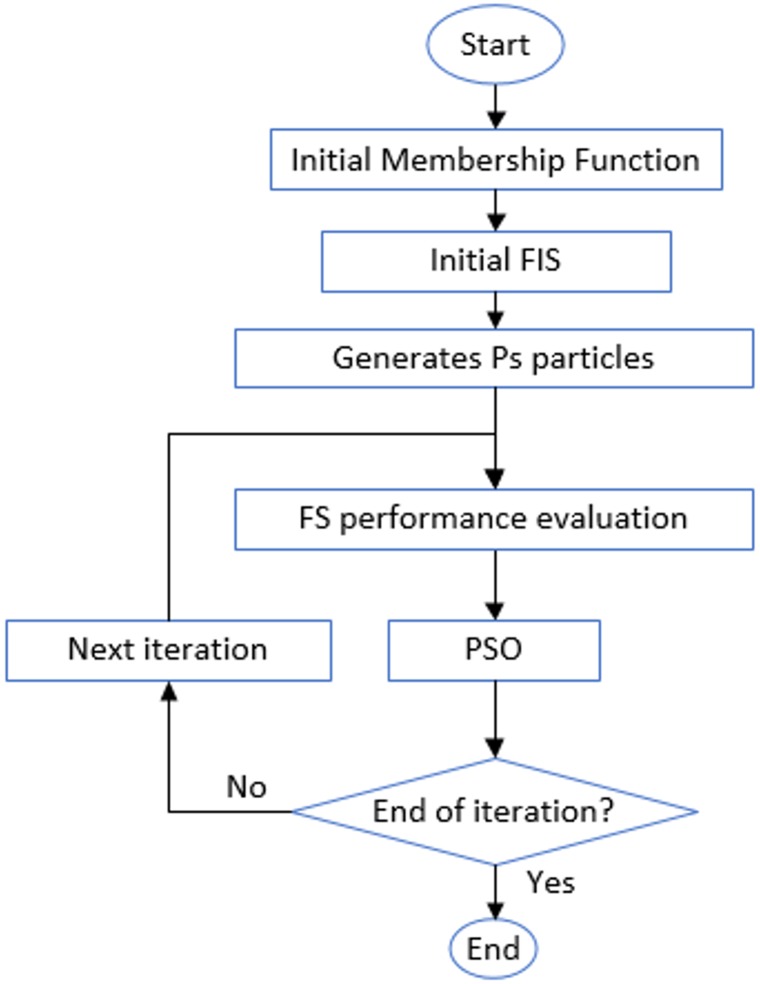
Diagram of sequential combination of PSO and ANFIS.

Fig 8 depicts the diagram of the sequential PSO and ANFIS combination [73]. In PSO, swarm starts with a group of random solutions, each of which is called a particle, and $\vec{s_{i}}$ represents the particle’s position. Likewise, a particle swarm moves in the problem space, where $\vec{v_{i}}$ expresses the particle’s velocity. A function *f* is evaluated at each time step through input $\vec{s_{i}}$. Every particle records its best position related to the best fitness gained to this point, in $\vec{p_{i}}$ vector. $\vec{p_{i}^{g}}$ tracks the most appropriate position identified by any neighborhood member. In universal version of PSO, $\vec{p_{i}^{g}}$ represents the most appropriate point in the entire population. A new velocity is achieved for any particle *i* in each iteration according to the best positions of individual, $\vec{p_{i}\left(t\right)}$, and ${\vec{p}}_{i}^{g}\left(t\right)$ neighborhood. The new velocity can be presented by:

$$\vec{v_{i}}\left(t+1\right)=w\vec{v_{i}}\left(t\right)+c_{1}\vec{\emptyset_{1}}.\left(\vec{p_{i}}\;\left(t\right)-\vec{x_{i}}\;\left(t\right)\right)+c_{2}\vec{\emptyset_{2}.}\left(\vec{p_{i}^{g}}\;\left(t\right)-\vec{x_{i}}\;\left(t\right)\right)$$ (10)

In Eq 10, *w* represents the inertia weight. The positive acceleration coefficients are shown by *c*_1_ and *c*_2_. $\vec{\emptyset_{1}}$ and $\vec{\emptyset_{2}}$ represent uniformly-distributed random vectors in [0,1], in which a random value is tried for every dimension. $\vec{v_{i}}$ limit in the [-$\vec{v_{max}}$, $\vec{v_{max}}$] series is reliant on the problem. Provided that the velocity exceeds the mentioned limit, in some cases it is rearranged within its suitable limits. The position of every particle alters depending upon the velocities as follows

$$\vec{s_{i}}\left(t+1\right)=\vec{s_{i}}\left(t\right)+\vec{v_{i}}\left(t+1\right)$$ (11)

According to Eqs 10 and 11, the particles incline to gather nearby the best. PSO use for designing a FS, or parameter optimization is expressed as:

$$R_{i}:if\;x_{1}\left(k\right)\;is\;A_{i1}\;And\ldots And\;x_{n}\left(k\right)\;is\;A_{in}\;,\;Then\;u\left(k\right)is\;a_{i}$$ (12)

Here, *α*_*i*_ is a crisp value, *k* represents the time step, the input variables are *x*_1_(*k*), …, *x*_*n*_(*k*), *A*_*ij*_ is a fuzzy set and *u*(*k*) signifies the output variable for system.

For the FS in Eq 12 which comprises *r* rules and *n* input variables, its free parameters are defined through a position vector:

$$\vec{s}=\left[m_{11},b_{11},\ldots ,m_{1n},b_{1n},a_{1},\ldots \ldots ,m_{r1},b_{r1},\ldots ,m_{rn},b_{rn},a_{r}\right]\in {ℜ}^{D}$$ (13)

$$m_{rj}=x_{j}\left(k\right),\;b_{rj}=b_{fix},\;j=1,\ldots ,n$$ (14)

Following the process of rule creation and initialization, the preliminary antecedent part parameters are outlined. According to Eqs 13 and 14, the *i*th solution vector $\vec{s_{i}}$ is created as:

$$\vec{s_{i}}=\left[s_{i1}\;s_{i2}\ldots \;s_{iD}\right]=[m_{11}+\Delta m_{11}^{i},b_{fix}+\Delta b_{11}^{i},\ldots ,m_{1n}+\Delta m_{1n}^{i},b_{fix}+\Delta b_{1n}^{i},a_{1},\ldots ,m_{r1}+\Delta m_{r1}^{i},b_{fix}+\Delta b_{r1}^{i},\ldots ,m_{rn}+\Delta m_{rn}^{i},b_{fix}+\Delta b_{rn}^{i},a_{r}]$$ (15)

In the equation, Δ*m*_*ij*_ and Δ*b*_*ij*_ signify the numbers of small random, *α*_*i*_ designates a random number distributed arbitrarily and homogeneously in the fuzzy system output range. The evaluation function *f* for $\vec{s_{i}}$ is calculated based upon the fuzzy system performance in Eq 15.

PSO looks for the best originator part parameters. *P*_*s*_ represents the population size. Eq 4 sets the elements in position $\vec{s_{i}}$. When *t* = 0, the $\vec{s_{1}}\left(0\right),\ldots ,\vec{s_{p}}\left(0\right)$ or initial positions are created arbitrarily according to the best-performing FS found in ACO (${\vec{s}}_{PSO}$). $\vec{s_{1}}\left(0\right)$ is considered similar to ${\vec{s}}_{PSO}$. The left *P*_*s*_ − 1 particles, $\vec{s_{1}}\left(0\right),\ldots ,\vec{s_{p}}\left(0\right)$, are created by addition of uniformly-distributed random numbers to ${\vec{s}}_{PSO}$ shown as:

$$\vec{s_{i}}\left(0\right)={\vec{s}}_{PSO}+\vec{w_{i}},\;i=2,\ldots ,P_{s}$$ (16)

$\vec{w_{i}}$ represents a random vector. The primary speed values of all particles, $\vec{v_{i}}\left(0\right),\;\;i=1,\ldots ,P_{s}$, are generated randomly. Each particle’s performance is evaluated according to the FS it signifies. *f* is described as the *E*(*t*) or error index mentioned above. The best position ($\vec{p_{i}}$) of each particle and the best particle $\vec{p_{g}^{i}}$ in the whole population is obtained according to *f*. Eqs 10 and 11 overhaul the velocity and position of each particle. The whole learning procedure is accomplished as soon as a pre-defined paradigm is obtained [73].

There are five PSO main parameters used during conducting experiment as shown in Table 3, which are maximum number of iterations, population size of the domain, inertia weight damping ratio and inertia weight, global learning coefficient and personal learning coefficient. For this case study, we determined these parameters optimum values by trial and error procedure.

**Table 3:** Parameter characteristics used in this study.

Population Size	Iterations	Inertia Weight	Damping Ratio	Learning coefficient	
				Personal	Global
40	1000	1	0.99	1	2

### Evaluation of model performances

Statistical tests offer a certain level of assurance about the validity, non-randomness of the [74]. Specifically, in this paper, we used root mean square error (RMSE), Eq 17 and coefficient of determination (R^2^), Eq 18 to compare forecasting errors of between different models and determine the proportion of the variance of one variable that is predictable from the other variable, respectively.

The following are the statistical indicators adopted to examine the ANFIS model performance:

1. root-mean-square error (RMSE)
  $$RMSE=\;\sqrt{\frac{\sum_{i=1}^{n}{\left(O_{i}-P_{i}\right)}^{2}}{n}}$$ (17)
2. Coefficient of determination (R^2^)
  $$R^{2}=\;\frac{{\left[\sum_{i=1}^{n}\left(O_{i}-{\bar{O}}_{i}\right).\left(P_{i}-{\bar{P}}_{i}\right)\right]}^{2}}{\sum_{i=1}^{n}\left(O_{i}-{\bar{O}}_{i}\right).\sum_{i=1}^{n}\left(P_{i}-{\bar{P}}_{i}\right)}$$ (18)
  *n* is the total number of test data, *P*_*i*_ = measurement values and *O*_*i*_ = ANFIS value.

## Evaluations

### Simulation findings

**Fig 9:**
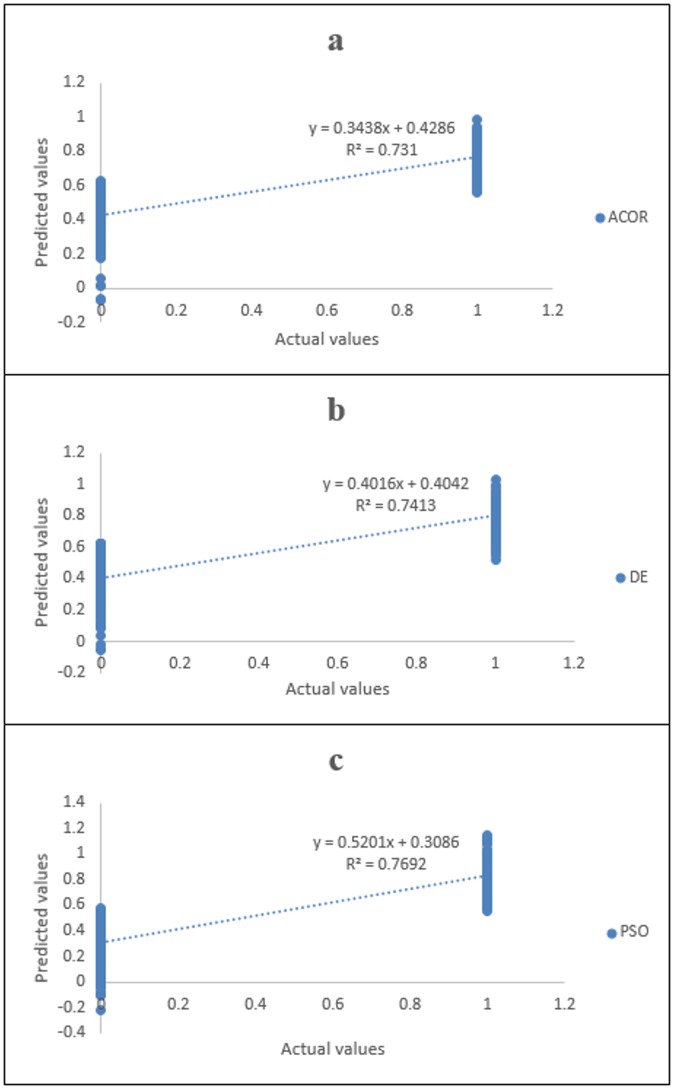
Performance of ANFIS-DE, ANFIS-ACO and ANFIS-PSO for estimation of mobile malware. (a) ANFIS-DE fit line. (b) ANFIS-ACO fit line. (c) ANFIS-PSO fit line.

The preliminary data aids in creating the hybrid soft computing method, and three methods were principally used to predict the data. The scatterplot in Fig 9 shows the estimation of best mobile malware parameters. Next, the fit line with equation *y* = *α0 + αl* was generated.

### Performance analysis

**Fig 10:**
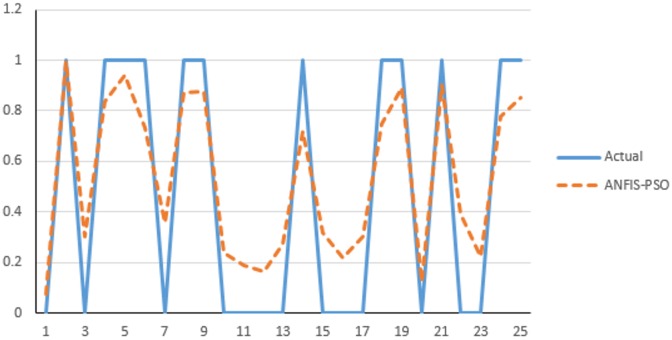
Prediction of the optimum parameters of mobile malware analysis by ANFIS-PSO for testing data.

The available experimental data were used for assessing the performance of methods and identifying importance of the parameters. The R^2^ and RMSE were used to make comparison between the real and predicted values for the soft computing method. Tables 4 and 5 present the summary of comparison between ANFIS-DE, ANFIS-PSO and ANFIS-ACO. The performance analysis prediction of mobile malware using ANFIS-PSO is presented in Fig 10.

**Table 4:** Analysis of Mean Square Error (MSE) and Standard Deviation (StD) for different methods.

		ANFIS-ACO	ANFIS-DE	ANFIS-PSO
*Training Data*				
	MSE	0.20948	0.19487	0.18605
	StD	0.4577	0.44144	0.43133
*Test Data*				
	MSE	0.21098	0.19576	0.18581
	StD	0.45932	0.44245	0.43107

**Table 5:** Analysis of performance for different methods to identify the optimum parameters of a mobile malware prediction model.

Method	Training		Testing	
	Error (RMSE)	Coefficient of determination (R^2^)	Error (RMSE)	Coefficient of determination (R^2^)
ANFIS-PSO	0.43133	0.7692	0.43106	0.7721
ANFIS-ACO	0.45769	0.7311	0.45932	0.7392
ANFIS-DE	0.44144	0.7413	0.44244	0.7562

**Fig 11:**
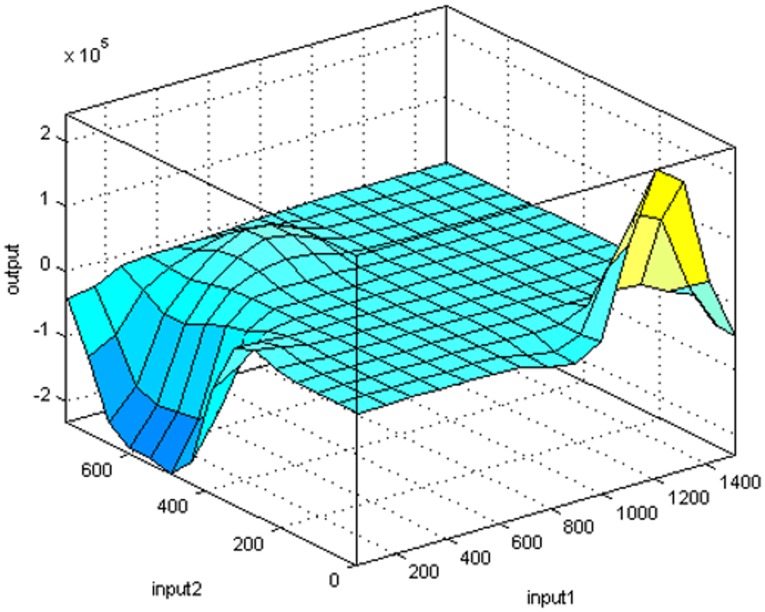
ANFIS-PSO decision surface for the detection model: input1—Maximum_Frame, input2—Frame_STD.

The ANFIS-PSO decision surface for mobile malware detection is shown in Fig 11 for the two extracted parameters, Maximum_Frame and Frame_STD.

**Fig 12:**
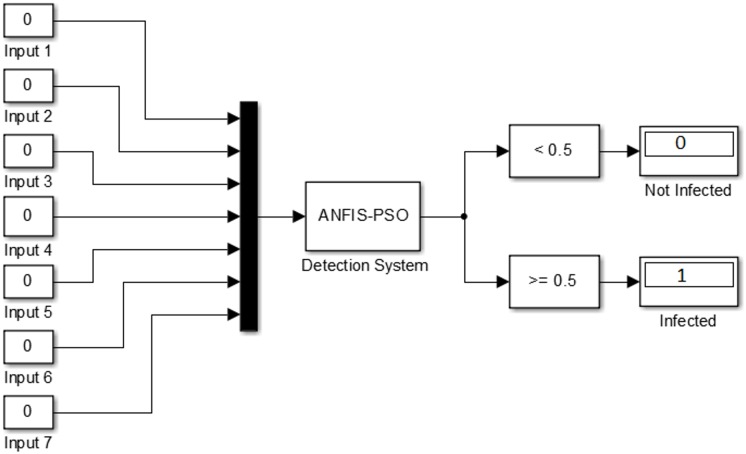
SIMULINK block diagram for ANFIS-PSO detection of android mobile malware.

It can be noted from Fig 10, when the model output is smaller than 0.5, the decision should be uninfected and when the model output is larger than 0.5 the decision should be infected. Finally based on this observation, we created SIMULINK block diagram for ANFIS-PSO detection of android mobile malware Fig 12.

## Conclusion

The number and sophistication of Android malware are increasing and evolving, which necessitates the development of more effective malware detection systems. Recent advances in the literature suggests that artificial intelligence techniques are a promising approach to detect mobile malware. Generally, mobile malware communicates with a compromised server or a server under the control of an attacker, via a network. Thus, in this work, we focused on network based features. Specifically, application traffic was filtered for its parameters and calculation was performed on these parameters to obtain the required features. We also proposed three system agents to capture and manage the pcap file for the data preparation phase to improve our detection system in terms of efficient data processing.

It is known that ANFIS scheme is computationally efficient and well-adaptable with optimization and adaptive techniques. This scheme can also be combined with expert systems and rough sets for other applications, as well as used with other systems to handle more complex parameters. Another advantage of ANFIS is its speed of operation, which is much faster than in other control strategies. The laborious task of training membership functions is performed in ANFIS using metaheuristic optimization algorithms (due to the nature of fuzzy systems).

A novel hybrid method (integrating ANFIS and PSO) was proposed in this study to forecast the best parameters of a mobile malware analysis. The ANFIS-PSO is compared with two hybrid optimization approaches, namely: ANFIS-ACO and ANFIS-DE. Our findings demonstrated the utility of the proposed method. For example, ANFIS-PSO outperforms other approaches with RMSE, 0.43133 in training and 0.43106 in testing. Its coefficient of determination (R2) also achieves an improved performance (e.g. 0.7692 in training and 0.7721 in testing). For example, a majority (77%) of the variations in predicted result can be explained by the linear relationship between actual value and predicted model. This suggests that the prediction model has a strong positive linear correlation in terms of its accuracy in predicting and detecting Android malware.

Future work includes extending the research to a refined selection of variables (e.g. due to evolution of malware). Another potential research area is to address the known challenges in the selection of input variables, such as identifying and discarding irrelevant variables (noise). It is, therefore, useful to design methods that require reduced number of input variables (i.e. reducing the complexity of the model) yet achieving better efficiency and accuracy.
